# A Brief Survey of Telerobotic Time Delay Mitigation

**DOI:** 10.3389/frobt.2020.578805

**Published:** 2020-12-15

**Authors:** Parinaz Farajiparvar, Hao Ying, Abhilash Pandya

**Affiliations:** Electrical and Computer Engineering, Wayne State University, Detroit, MI, United States

**Keywords:** teleoperation, Robotics, telesurgery, time series prediction, machine learning, recurrent neural network, Long Short-Term Memory, Sequence to sequence model

## Abstract

There is a substantial number of telerobotics and teleoperation applications ranging from space operations, ground/aerial robotics, drive-by-wire systems to medical interventions. Major obstacles for such applications include latency, channel corruptions, and bandwidth which limit teleoperation efficacy. This survey reviews the time delay problem in teleoperation systems. We briefly review different solutions from early approaches which consist of control-theory-based models and user interface designs and focus on newer approaches developed since 2014. Future solutions to the time delay problem will likely be hybrid solutions which include modeling of user intent, prediction of robot movements, and time delay prediction all potentially using time series prediction methods. Hence, we examine methods that are primarily based on time series prediction. Recent prediction approaches take advantage of advances in nonlinear statistical models as well as machine learning and neural network techniques. We review Recurrent Neural Networks, Long Short-Term Memory, Sequence to Sequence, and Generative Adversarial Network models and examine each of these approaches for addressing time delay. As time delay is still an unsolved problem, we suggest some possible future research directions from information-theory-based modeling, which may lead to promising new approaches to advancing the field.

## 1. Introduction

Teleoperation or telerobotics is a broad area in robotics with a long and rich history which has been a major area of interest over the last decade with numerous applications. This form of robotics has a user at a local (master) location controlling a robot at a remote site (slave) with feedback (usually video) from that remote location. Goertz and Thompson (1954) started managing radioactive material with the help of mechanically built teleoperators and pioneered modern teleoperation in the 1950s. Interest in teleoperation has recently surged with critical applications in many domains, especially medicine (Sanchez et al., 2012; Livatino et al., 2014). The recent Covid19 pandemic has heightened the need for remote operations for both medical interventions and other logistics (Yang et al., 2020).

**Figure 1:**
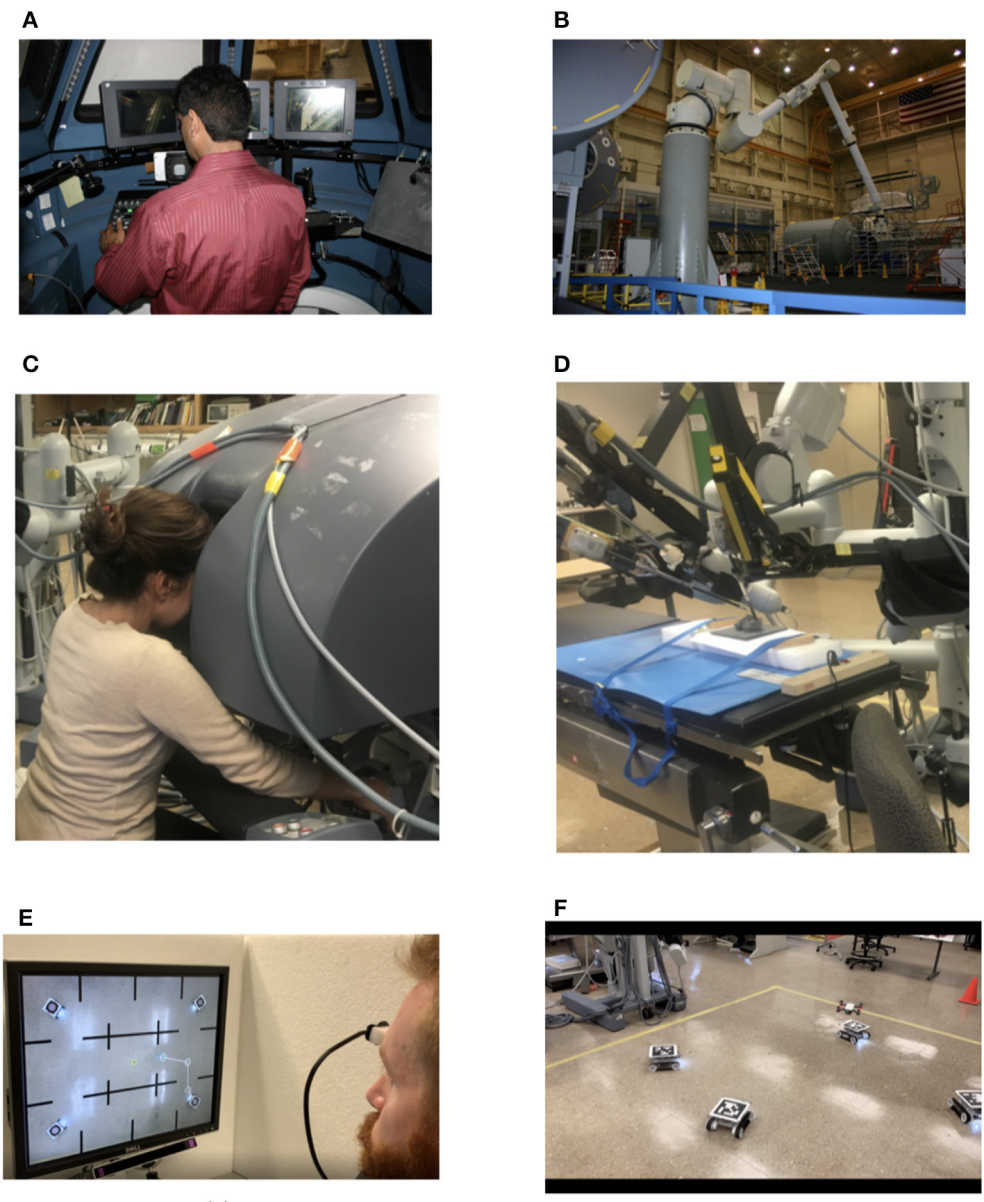
Different applications of teleoperation. **(A)** Operator of a space robot arm, **(B)** the space robot arm (Chintamani et al., 2009). **(C)** Telesurgery with a research da Vinci surgical system (Eslamian et al., 2020), surgeon side. **(D)** Remote patient side, **(E)** ground robot control with aerial view (Lucas et al., 2012)—operator controls ground robots using eye-tracking and **(F)** the ground robots (Lucas et al., 2012).

Examples of useful recent applications of teleoperation range from space operations, military (Chen, 2010), underwater exploration (Saltaren et al., 2007), mining, nuclear/toxic material handling, military, and robotic-assisted medical interventions (Madder et al., 2019). Figure 1 shows three applications from our laboratory, which includes three remote users of medical, space, and ground robots. A recent paper in medical robotics (Madder et al., 2019) studied the effect of time delay on a robotic coronary telestenting system. They use a robotic system at distances over 100 miles and simulate network latency from 0 to 1,000 ms. They show that 400 ms latency is acceptable (they are able to perform stents), delays between 100 and 250 ms did not make a significant difference from no delay, and more than 400 ms delay affected the surgeon's performance. More investigation on the effect of time delay on the surgeon's performance in the medical teleoperation systems is explored by Rosen and Hannaford (2006) and Lum et al. (2009). Also, Orosco et al. (2020) found that negative motion scaling (less remote instrument movement for a particular master controller movement) improved performance for time-delayed robotic surgery.

Several recent review papers on the topic of teleoperation with time delay have been published. Sun et al. (2014) review wave variable control methods, which are an extension of passivity theory. They highlighted the issues of wave reflection and drift as barriers to this approach. Muradore and Fiorini (2016) review bilateral teleoperation algorithms where haptic feedback is difficult with a time-delayed interface. They review algorithms that lead to the stability of haptic interfaces based on passivity theory. Uddin and Ryu (2016) survey predictive control approaches to mitigate time delay. They cover model-based approaches mostly related to predictive control. Kebria et al. (2018) review internet-based teleoperation systems. They deal with issues of delay, packet loss, jitter, and blackout seen in internet-based approaches and cover traditional and adaptive-based control approaches, neural networks, and fuzzy-based approaches where they model the uncertainty by modeling time delay.

In this paper, we review the trends and strategies used to solve the time delay issues related to teleoperation. First, we will briefly review some of the early approaches and examine predictive and statistical approaches. Then we explore the closely related field of time series prediction using machine learning and AI techniques and how they have been or can be applied to this field. We will also provide our analysis and projection for open research questions and on future research directions that can utilize the recent explosion and progress in deep neural networks, machine learning, and information theory.

In section 2, we review the early approaches for the time delay in teleoperation, which consist of primarily control theory approaches. In section 3, we introduce the time series prediction problem and highlight the similarities between the time delay problem and the general time series problem. In this section, we review statistical models and Neural Networks (NN) based methods. Finally, in section 4, we compare and discuss the methods and conclude with some future avenues of research and new ideas to solve time delay in teleoperation.

## 2. Traditional Approaches to Mitigating Time Delay in Teleoperation

Early research in the field deals primarily with creating appropriate user interfaces for teleoperation (Sheridan, 1992) and issues related to the communication channels between the remote and local sites. Due to distance, low speed, poor quality of the communication channels, and time delay, data corruption often occurs in teleoperation systems. The main issue that is researched in the literature is the time delay of communication channels. This is a fundamental problem related to signal transmission's physical limits and not necessarily on the hardware. Data transmission delay in teleoperation can be between less than a few milliseconds to many minutes based on the distance between the master and slave locations and the communication medium. Excessive delays can make teleoperation very difficult to perform. Research techniques to stabilize and mitigate time delay issues have been a significant area of interest in teleoperation.

### 2.1. Defining a Model for Time Delay

Ferrell and Sheridan's experiments (Ferrell, 1965) determined the impact of time delay on human operator performance in teleoperated manipulators. They used the servo-driven manipulator with two parallel slave fingers controlled by a human operator (master). In this experiment, the time delay is added to command signals from the human operator before it is received by the slave. Their research showed that operators respond to the delay with a move and wait strategy. That means that the operator moves the joystick and waits for feedback before responding again to the remote robot. Then the user starts a corrective step and waits again to recognize the remote system's delayed reaction and repeats the cycle until the operation is complete. Based on Hokayem and Spong (2006), the completion time of an assignment is defined as follows:

(1) $$\begin{array}{l} t\left(I\right)=t_{r}+\overset{N\left(I\right)}{\underset{i=1}{\sum }}\left(t_{mi}+t_{wi}\right)+\left(t_{r}+t_{d}\right)N\left(I\right)+t_{g}+t_{d}, \end{array}$$

where *I* is the measure of difficulty, *N*(*I*) is the number of the movements, *t*_*r*_ is the human's reaction time, *t*_*mi*_ is the movement duration, *t*_*wi*_ is the waiting time after each move, *t*_*g*_ is the grasping time and *t*_*d*_ is the delay time introduced into the communication channel. The completion time for a particular assignment depends linearly on the delay factor in the control loop; therefore, the longer the delay, the greater the completion time. Ferrell and Sheridan's experiments (Ferrell, 1965) conclude that the move and wait strategy works; however, it takes a longer time and has smoothness implications.

### 2.2. Supervisory Control Methods

Supervisory control with a direct connection between the master and remote sides was introduced by Ferrell and Sheridan (1967), to address the time delay problem. In supervisory control, based on the difficulty of the task and the order of the autonomy of the process, the control could be either of symbolic or analog nature. In the symbolic approach, they introduce small autonomous sub-tasks, which are high-level commands used for the local controller. Whitney (1969) introduced the supervisory methodology from an optimization perspective by developing a discrete-state-space and applying search strategies to accomplish the ideal performance of the task.

With the advancement in microprocessor design and programming, yet another solution was introduced for the time delay in teleoperation. In this approach, a processor (at the remote site) was able to do simple tasks like close the gripper or move it from point A to point B. Other complex tasks could be performed by chaining the simple commands together. In this way, only high-level task commands need to be sent. Modular software for simple or repetitive tasks was introduced in this area and improved the performance and minimized communication time by using inter-processor communication and control mode selection (Fong et al., 1986). Task-Oriented Supervision Command System (Madni et al., 1983), and the language-aided robotic teleoperation system in Sato and Hirai (1987) provide a method to do simple tasks. In addition, visual models helped control process by adding graphics models of the motion of the robot (Stark et al., 1987; Hirzinger et al., 1989; Bejczy and Kim, 1990). Bejczy et al. (1990) used the phantom robot models to predict real robot motion. Buzan and Sheridan (1989), applied a predictive operator aid to handle the time delay in telemanipulator systems.

### 2.3. Predictive Control-Based Approaches for Time Delay

Time delay is a challenge when designing stable controllers. Control solutions for time-delayed teleoperation systems open up new questions for the interpretation of the fundamental principles of control systems and render it necessary to re-evaluate these ideas. In many instances, traditional techniques fail to stabilize systems, especially when the time delay varies or blackout occurs. The impact of time delay on the stability of a remotely controlled system was studied by Varkonyi et al. (2014). The authors consider a remotely controlled system where keeping the system stable with time delay was a challenge. Varkonyi et al. (2014), they showed that traditional control system methods are not successful when there is a variable time delay or communication blackout.

A good review, Uddin and Ryu (2016), provides a comprehensive comparison and analysis of different approaches for predictive control. This paper covers mostly model-based approaches for teleoperation. A multi-model predictive controller was proposed by Sirouspour and Shahdi (2006). They propose a discrete linear quadratic Gaussian (LQG) controller for teleoperation with time delay in communication (Sirouspour, 2005). In this case, the sampling rate was restricted as the delay increases in order to decrease the computational load and prevent potential numerical issues. This method has limits as the closed-loop reaction and teleoperation stability can be adversely affected by a small sampling rate.

Sirouspour and Shahdi (2006) propose a novel approach to the reduction and output-feedback control of MIMO (multiple-input and multiple-output) systems with nonidentical delay and they show that this system acquires the detectability and stabilizability properties of the original system. Their second achievement in that paper was that they formulate teleoperation under delay as a multi-model continuous-time LQG synthesis problem using the proposed output-feedback control approach.

### 2.4. Passivity-Based Methods

It is possible to model the delay problem mathematically as a “passivity-based teleoperation.” Desoer and Vidyasagar (1975) introduce passivity-based teleoperation in bilateral teleoperation to ensure stability and performance with packet loss and time delay. There are different passivity-based approaches to model the master-slave teleoperator system, such as 2-port networks (Buzan and Sheridan, 1989), impedance matrix (Raju et al., 1989), hybrid matrix (Hannaford, 1989), scattering approach (Anderson and Spong, 1992), constant time delay (Anderson and Spong, 1992), scaling (Colgate, 1991), wave variables (Niemeyer and Slotine, 1991a,b; Benedetti et al., 2001; Ganjefar et al., 2002; Munir and Book, 2002), and geometric scattering (Stramigioli et al., 2002). Hokayem and Spong (2006) examined the theoretical control approaches to address the time delay problem and information loss. In passivity-based teleoperation, researchers represent the master/slave teleoperation system with linear models. Nuño et al. (2011) review several passivity-based controllers for non-linear bilateral teleoperation. Polat and Scherer (2012) presents stability analysis for uncertain bilateral teleoperation systems using the IQC framework. They formulate stability using network theory and run numerical test cases to verify their formulation. In addition, Tugal et al. (2016) investigate the stability of passive multipliers and Zames-Falb multipliers with the IQC framework for both time-invariant and time-variant time delays.

Another kind of teleoperation that was introduced is internet-based teleoperation (Goldberg et al., 1995; Kebria et al., 2018). Different approaches to deal with random time delay in message transmission over the internet have been researched. This area of real-time communication for teleoperation over the internet has been active since the 1990s. Xi and Tarn (2000) propose non-time referenced action control method to deal with random time delay, and Oboe (2003) show the success of real-time closed-loop control systems for telerobotics over the internet.

Furthermore, predictive techniques are introduced to model and mitigate the random time delay with fuzzy adaptive control methods by Lu et al. (2017) and Mirfakhrai and Payandeh (2002) where they used an autoregressive model. Ye et al. (2002) and Shen et al. (2019) used nonlinear time series analysis to understand the delay behavior and estimate time delay. The application of neural networks for creating a robust teleoperation system with time-varying communication delay was studied by Li and Su (2013). These papers opened up a new area of research with time series analysis/prediction. We will explain time series prediction and the different avenues of research in the next section.

In summary, control-based methods to mitigate time delay for teleoperation systems can be divided into two approaches. In the first approach, guaranteeing stability is used to deal with variable time delay. In addition, Passivity-based control methods are applied for modeling master-slave teleoperator systems. A second approach is a predictive approach, which consists of model-based approaches like the LQG controller (Sirouspour, 2005; Sirouspour and Shahdi, 2006). This approach has shown improvement in performance in terms of stability and transparency. Another example of this approach is when a processor performs complex tasks by chaining simple commands together (Hirzinger et al., 1989). That method could take advantage of a time series prediction to further mitigate time delay.

## 3. Time Series Prediction Methods for Mitigating Time Delay in Teleoperation

An avenue of research in the time delay problem in teleoperation is using time-series predictions, where time-series predictions are used to compensate for the time delay. In time series prediction, the goal is to predict future values based on past observations which consist of intrinsic patterns. To determine a model that expresses the pattern of time series, we need a method to describe the important features of the time series pattern and explain how the past affects the future or how two-time series can “interact.”

In general, time series have three major common patterns:

1. **Trend**: The specific direction of the time series can be a long-term increase or decrease in the data (Parmezan et al., 2019).
2. **Seasonality**: The repetitive patterns at predictable intervals.
3. **White Noise**: The unpredictable fluctuation with no seasonality or trend.

**Figure 2:**
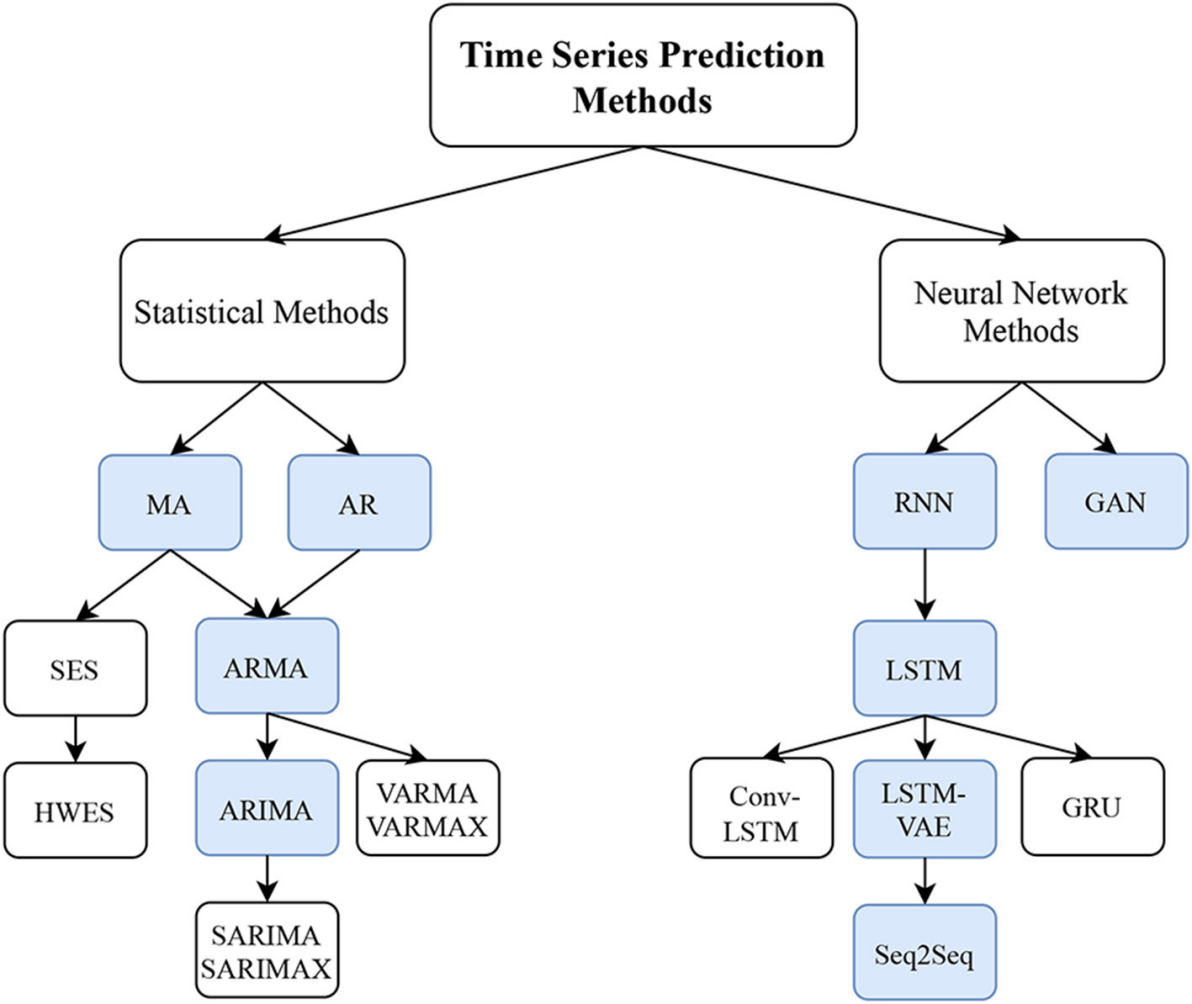
Hierarchical structure of time series prediction techniques in both statistical and machine learning approaches. For statistical methods, our main focus is on the blue boxes (i.e., *AR, MA, ARMA*, and *ARIMA*) as they are the more fundamental methods. The remaining methods are their extensions. For Neural Network Methods, we focus on the *RNN, LSTM, Seq2Seq*, and *GAN* methods, which are in blue boxes.

In this paper, we divide time series prediction methods into two main methods: (as shown in Figure 2): **Statistical approaches**, **Neural Network approaches**. We cover these two methods as recent approaches for time series prediction.

### 3.1. Examples of Time Series Prediction in Delay Mitigation

Several early approaches for delay mitigation used time series prediction. Mirfakhrai and Payandeh (2002) employed an autoregressive model to forecast future values of time delay. The predictions were used with a look-up table to tune the system by adjusting the gain and decreasing the mismatch between forces and velocities at the master and slave sides. In their study, the slave side is assumed to be precisely similar to the master side; also, the slaves' delay and master communication are equal, and there is no scaling between the master and slave. Ye et al. (2002) studied the delay for round trip time (RTT) in internet-based communication. They use a linear correlation of RTT by computing the autocorrelation and power spectrum. In this paper, the authors used the maximum entropy principle (MEP) which is a linear algorithm to predict one step ahead of the RTT value.

Recently, there have been several papers that use time-series predictions for teleoperation time delay mitigation. For instance, Chen et al. (2020) utilizes a statistical approach using a multivariate linear regression model to forecast time delay for space teleoperation. When time delay is known in advance, control of remote systems is improved in comparison with the previous approaches that included sparse multivariate linear regression (SMLR) (Chen et al., 2019), autoregressive (AR), neural network (NN), and cubic polynomial model-based (CPMB) approaches. Su et al. (2020b) apply deep convolution neural networks to identify a robot tool's dynamics for bilateral teleoperation. Belhaj and Tagina (2009) apply RNNs to model and predict internet end-to-end time delay. Su et al. (2020a) propose an improved RNN to predict the trajectory of manipulators. Aburime et al. (2019) applied recursive least squares filtering to identify the delay and target waypoints. They were able to show that an aerial vehicle can estimate a waypoint based on an appropriate filter selection while it also monitors the user commands. Zheng et al. (2019) also use a similar approach for a ground vehicle heading prediction. They use a blended approach with a Taylor series expansion with estimated noise to model the heading for remote-controlled vehicles. They use the delayed signals to predict the heading of the vehicle such that in the event of a delay, the vehicle does not go off course. Arita and Suzuki (2019) employ an exponential statistical prediction model of human gaze points to assist in the maneuvering of teleoperated robots. They use a first-order gaze movement model with a time delay to predict specific target points and use these as points of movement during the time delay. Jung et al. (2019) utilize a predictive display using a kinematic model of the human head and neck along with camera parameters. This is an interesting approach where they use the relationship between camera orientation and the human controllers' positional changes to predict future images based on current images using a simple linear model of motion.

### 3.2. Statistical Models for Time Series Prediction

In this subsection, we discuss the statistical methods for time series prediction. We also explain the use of statistical models to try and predict the near-term future to help mitigate time delay. This approach uses predictions based on statistical methods to model incoming data with the aim of predicting the next steps and replacing the data which is not received because of time delay.

Statistical models summarize the data with equations that represent a relation between input and output (Wasserman, 2013). A predictive statistical model defines a process on data to predict new or future observations (Shmueli, 2010) based on the observed data. The evolution of statistical techniques started with a simple technique of linear auto-regression (AR) (Mills and Mills, 1991). This technique uses a model that predicts future steps based on a linear model of the past data. Similarly, some techniques use a moving average (MA) as a predictor of future events (Box and Pierce, 1970). This technique also uses a stochastic component that models noise in the system. Both the AR and MA techniques were combined to create a system that was named ARMA. In this method, predictions were based on both a linear regression as well as a moving average. The next evolution added a nonlinear component (integration) to the formulation and was named ARIMA. The addition of the integral term allowed better prediction of non-stationary signals (Mondal et al., 2014). Simple Exponential Smoothing (SES) is an extension of the MA method. It uses an exponential window function, which exponentially weights previous observations in the time series. It differs from MA which treats all previous steps equally. Holt Winter's Exponential Smoothing (HWES) is a triple exponential smoothing method that predicts the next time steps based on three factors—the weighted prior time step, the trend and the seasonality (Kalekar, 2004). Vector Autoregression Moving-Average (VARMA) is the generalization of the ARMA model to forecast a multivariate time series. Vector Autoregression Moving-Average with Exogenous Regressors (VARMAX) is the extension of the VARMA model along with the exogenous variables or covariates as the parallel independent input sequences (Poskitt, 2016). Seasonal ARIMA or Seasonal Autoregressive Integrated Moving-Average (SARIMA) is an extended version of ARIMA with the ability to capture the seasonality of the time series (Hyndman and Athanasopoulos, 2018) and Seasonal Autoregressive Integrated Moving-Average with Exogenous Regressors (SARIMAX) is a method with exogenous data to improve the prediction result. In the next section, we will cover the details of these four methods: AR, MA, ARMA, ARIMA. All the other methods are extensions of these four methods and shown in Figure 2 as white boxes. The details of these extensions are not covered extensively as they are closely related to the core methods.

#### 3.2.1. AR Model

Auto-regressive (AR) models describe the linear dependency of the previous values to the predicted values. The basic components of an AR model include linear coefficients for prediction, a model of error (noise) in the system, and a method to determine the extent of the past data needed for prediction based on an autocorrelation function. The first-order Auto-regressive model AR(1) is the linear model between the value of time step at time *x*_*t*_ and previous time step *x*_*t*−1_ which includes coefficients (ϕ_0_, ϕ_1_) with assumption |ϕ_*i*_| < 1, where *i* = 1, 2 and noise at time *t*, ω_*t*_ is defined as:

(2) $$\begin{array}{l} x_{t}=\phi_{0}+\phi_{1}x_{t-1}+\omega_{t} \end{array}$$

where $\omega_{t}\sim N\left(0,\sigma_{\omega }^{2}\right)$ (i.e., Gaussian distribution with zero mean and $\sigma_{\omega }^{2}$ as a variance) is the present error and ϕ_1_ is the slope of AR(1).

In general an *AR*(*p*) model (where *p* is the order of the model which can be selected as a design parameter) is given by the following equation:

(3) $$\begin{array}{l} x_{t}=\phi_{0}+\phi_{1}x_{t-1}+\phi_{2}x_{t-2}+\cdots +\phi_{p}x_{t-p}+\omega_{t} \end{array}$$

Mirfakhrai and Payandeh (2002) and Hu et al. (2012) use this auto-regressive model to predict the value of time delay in the future. In their model *x*_*t*_ is the signal that is modeled and ω_*t*_ is the white noise with auto-correlation. To determine the order of the AR model researchers use the *Partial Autocorrelation Function (PACF)* (Mishra and Desai, 2005). The *PACF* shows the autocorrelation between the variable and a lag without considering the effect of lower-order lags. For example, when the PACF tends to zero at *k* lags, it shows the order of our AR model *p* is equal to *k*. Once an appropriate order has been determined, a model can be formed to allow prediction.

#### 3.2.2. MA Model

Another method for modeling the time series data is the moving average (MA) model. The MA model takes the average of the previous values of the time series to predict future values. The basic components of an MA model are similar to the AR model except that in this model, we want to know how much noise in the past affects the prediction. The first order of an MA model is denoted by MA(1) and is defined as:

(4) $$\begin{array}{l} x_{t}=\mu +\omega_{t}+\theta \omega_{t-1} \end{array}$$

where μ is the mean of the time series, θ is the parameter of the model and ω_*t*_ is the white noise with zero mean and $\sigma_{\omega }^{2}$ as a variance.

General MA(q) models:

(5) $$\begin{array}{l} x_{t}=\mu +\omega_{t}+\theta_{1}\omega_{t-1}+\theta_{2}\omega_{t-2}+\cdots +\theta_{q}\omega_{t-q} \end{array}$$

A property of MA(q) models in general is that there are nonzero autocorrelations for the first *q* lags and autocorrelations = 0 for all lags ≥ *q*. Similar to AR, in the MA model, we can find the order of the model based on the *Autocorrelation Function (ACF)*. The *ACF* shows the autocorrelation of a variable and a lag of itself (Moayedi and Masnadi-Shirazi, 2008). Once an appropriate order has been determined, a model can be formed to allow prediction.

#### 3.2.3. ARMA Model

The AR model is a prediction based on the lags of the data. The MA model is a prediction based on the past noise of the signal. In order to create a more general model that incorporates both these features, researchers combined these techniques (Choi, 2012). The combination of AR and MA models creates an autoregressive moving average model ARMA. There are three basic steps of creating an ARMA model. The first is, **model selection**, the second is **parameter estimation** and the third is **model checking** (Box et al., 2015). Model selection and parameter estimation are challenging problems in time series prediction. Rojas et al. (2008) introduced a method to determine the linear model automatically. An *ARMA(p,q)* model (where p and q are model orders) can be defined based on the AR(p) and MA(q) equations as follows:

(6) $$\begin{array}{l} x_{t}=\phi_{1}x_{t-1}+\cdots +\phi_{p}x_{t-p}+\omega_{t}+\theta_{1}\omega_{t-1}+\cdots +\theta_{q}\omega_{t-q} \end{array}$$

where the ω is the noise and the ϕ are the coefficients of AR and the θ are the coefficients of MA. The *ARMA* model can be used to predict stationary time series which have constant stochastic properties (mean, variance and correlation) with respect to time. Real-world time series data cannot be guaranteed to be stationary. Hence, a term needs to be added to make the model that can fit nonlinear non-stationary signals. Hua et al. (2013) employ this statistical method to estimate the time delay of a one-way internet connection.

#### 3.2.4. ARIMA Model

By adding a differentiation term (d) to the ARMA model, researchers converted the non-stationary signal to a stationary signal. The difference term added to a non-stationary signal makes it stationary, and this is commonly referred to as an integrated signal. Hence, this technique with the integrated signal addition is called the *ARIMA* model and is used for non-stationary time series prediction (Lorek and Willinger, 1996).

The *ARIMA(p,d,q)* model, is the combination of Autoregression, AR(p), integration, and Moving Average, MA(q). Here, *p* refers to the order of autoregression, *d* is the degree of difference and *q* is the order of the moving average terms.

In order to assess the trade-off between the *goodness of fit* and *over-fitting*, researchers have introduced a measure called the *Akaike Information Criterion* (AIC). AIC is used to compare the quality of statistical models. Based on Montgomery et al. (2015, Equation 2.44), AIC is used as a criterion to select the best ARIMA model parameters and is defined as:

(7) $$\begin{array}{l} AIC=-2L+\left(\log\left(n\right)+1\right)k \end{array}$$

where the *L* is the likelihood function logarithm, *n* is the number of observations, and *k* is the number of estimated parameters. A smaller AIC points to a better/more optimized model. AIC increases if the model is overfitting. Hence, AIC analysis allows us to balance our model between adding more parameters (at the cost of over-fitting) and better fitting.

Although ARIMA is the most general statistical model for forecasting in time series, it cannot deal with all nonlinear relationships like seasonality. Seasonal ARIMA or SARIMA is an extended version of ARIMA with this ability to capture the seasonality of the time series. There are various features of time series which can be analyzed with non-parametric (Aneiros-Pérez and Vieu, 2008) or hybrid models (Faruk, 2010). In addition, some machine learning algorithms were also introduced for the time series problem. The evolution of time series prediction using machine learning techniques has had several avenues of research and development in teleoperation and robotics. Yang et al. (2018) used a hidden semi-Markov model (HSMM) and Gaussian mixture models to improve the performance of teleoperation systems. In the following section, we see how this work was extended and review some neural network models for time series prediction.

### 3.3. Neural Network Methods for Time Series Prediction Problem

In this section, we examine various neural network-based methods. Prediction of individual sequences in terms of time or time series prediction is challenging and at the same time an important area of study in machine learning (Giles et al., 2001; Anava et al., 2013, 2015; Ak et al., 2015; Fang et al., 2017, 2020). Extracting good representative pairs of input and output data is essential in machine learning algorithms. These pairs are then used to train various types of neural network architectures. There are inherent properties of certain structures that make these predictions more accurate. Time series prediction uses models known as sequential data models. These sequential data models must maintain a particular order of the data streams.

Recurrent Neural Networks (RNNs) was among the first type of neural networks method for time series prediction (Mikolov et al., 2010). These networks then evolved to Long Short-Term Memory networks (LSTM) in order to model the past dependency in a more rigorous way (Hochreiter and Schmidhuber, 1997). A further refinement in neural network architecture was made with sequence-to-sequence (Seq2Seq) modeling. This architecture used LSTM in novel ways to improve time series prediction (Sutskever et al., 2014). Other neural network methods for time series prediction are Gated Recurrent Unit (GRU) (Cho et al., 2014), LSTM and Variational Auto-Encoder (LSTM-VAE) (Park et al., 2018), convolutional LSTM (convLSTM) (Karim et al., 2017), and Generative Adversarial Networks (GANs) for time series prediction (Yoon et al., 2019).

In the following section, we will review the basics of RNNs, LSTM, LSTM-VAE, Seq2Seq, and GAN techniques for time series prediction.

#### 3.3.1. RNN Model

**Figure 3:**
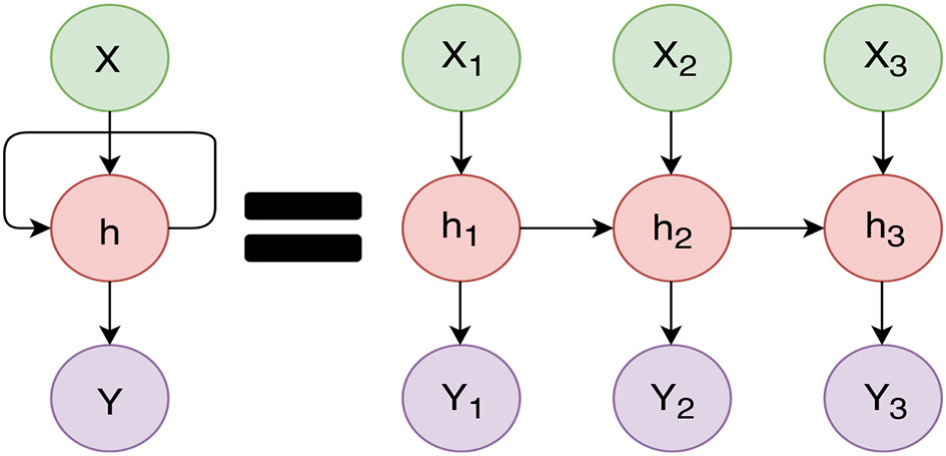
Recurrent Neural Network with feedback **(left)**. Unfolding of the Recurrent Neural Network **(right)**: The X values are the inputs (past values of the signal). Y values are the predictions. Each successive X is considered when predicting the next Y value. For instance *Y*_1_ is predicted based on *h*_1_, but *Y*_2_ is predicted based on both *h*_1_ and *h*_2_, and *X*_2_.

RNNs are different from feed-forward (traditional) neural networks in that they have a closed feedback loop and contain an element of memory. RNNs can remember the past with the use of a loop construct. This loop allows RNNs to persist the information. We can imagine the RNN as multiple copies of the same network, as shown in Figure 3. RNNs work well when we want to look at the recent information to predict the future. Connor et al. (1994) proposed a robust learning algorithm based on filtering anomalies from the data and used this filtered data for estimating parameters and do forecasting. In Giles et al. (2001), they examine the difficulties of RNNs for forecasting non-stationary and noisy data, and they introduced a pre-processing method to overcome these problems. However, when the gap between the relevant information and the prediction is large, RNNs become very slow and, in some cases, are unable to learn the long-term dependencies (Bengio et al., 1994).

#### 3.3.2. LSTM Model

**Figure 4:**
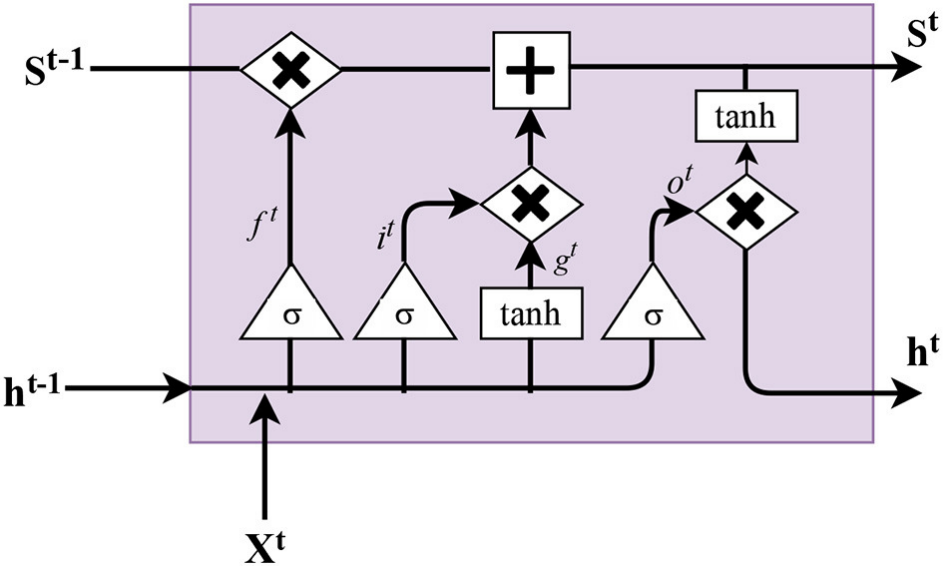
LSTM memory cell. Inputs: current input *x*^*t*^, state of previous time step *S*^*t*−1^ and output of previous time step *h*^*t*−1^. Outputs: updated state *S*^*t*^ and current output *h*^*t*^.

RNNs have issues with long-term dependencies and vanishing gradients. In order to deal with these issues (Hochreiter and Schmidhuber, 1997) introduced the LSTM networks (Figure 4). In this model, each ordinarily hidden layer is changed by adding a memory cell. We call these kinds of networks LSTM because it has long-term memory in terms of the weights and these weights can be altered by training. In addition, it also has short term memory (implemented by gates) in terms of a temporary activation (Lipton et al., 2015). Hence, in the LSTM, we have two main parts of the network the “forget gate” and “input gate.” The forget gates decide which information should be pushed away from the cell state, and the input gate decides which information needs to be stored in the cell state.

In each step, the forget gate considers the previous output *h*^(*t*−1)^ and input *x*^*t*^. The output of this forget gate is a number between 0 or 1. 1 represents “keep the value,” and 0 represents “forget value.” The following equation represents the forget gate:

(8) $$\begin{array}{l} f^{\left(t\right)}=\sigma \left(W_{f}\left[x^{\left(t\right)}+h^{\left(t-1\right)}\right]+b_{f}\right) \end{array}$$

where σ is the sigmoid function, *h*, *W*_*f*_, and *b*_*f*_ are hidden layer, weight and bias of the forget gate, respectively. In order to decide what part of the information we have to keep and use in the cell state at the next time step, we need the “input gate” *i*^*t*^ to specify which values we are updating. Then another gate with *tanh* as the activation function (shown by *g*^*t*^), is used to create a vector of the new candidate values to be added to the state cell *S*^*t*^. As a result, this method updates the new state based on multiplying the forget gate by the previous state and multiplication of input gate *g*^(*t*)^ as follow:

(9) $$\begin{array}{l} S^{\left(t\right)}=f^{\left(t\right)}⊙S^{\left(t-1\right)}+g^{\left(t\right)}⊙i^{\left(t\right)} \end{array}$$

where the ⊙ is element-wise multiplication. Finally, we have to determine the “output gate” *o*^*t*^, which relies on the previous output *h*^(*t*−1)^ and the input *x*^*t*^. Then the updated output *h*^*t*^ is calculated by multiplication of the “output gate” and *tanh* of the updated cell state as follows:

(10) $$\begin{array}{l} h^{\left(t\right)}=o^{\left(t\right)}⊙\tanh\left(s^{\left(t\right)}\right) \end{array}$$

There are a lot of extensions of the LSTM memory cells which are used for different time-series applications like convLSTM (Kim et al., 2010; Xingjian et al., 2015) for weather forecasting and video prediction (Lotter et al., 2016). Gated Recurrent Unit (GRU) is a simple version of LSTM (Cho et al., 2014). It combines the forget and input gate of the LSTM and reduces the complexity by decreasing the number of parameters.

#### 3.3.3. LSTM-VAE Model

Another framework of LSTM is the LSTM-VAE, which is a neural network based on both a variational autoencoder to compress the input and an LSTM for prediction. The encoder-decoder LSTM architecture for time series data is used by Park et al. (2018) for multi-modal anomaly detection for robot assistant feeding. Park et al. (2018) employed the LSTM unit for modeling the dependencies in the time series, as a variational autoencoder models the probability distribution of observations using variational inference (VI). LSTM has the advantage of both short and long term memory. However, in the case of multiple time series step predictions with dynamic output lengths, the system needs retraining to get new model parameters due to the requirement of different output lengths. In the next section, a new LSTM-based model called Seq2Seq is introduced. It can allow and improve dynamic multiple time series prediction.

#### 3.3.4. Seq2Seq Model

**Figure 5:**
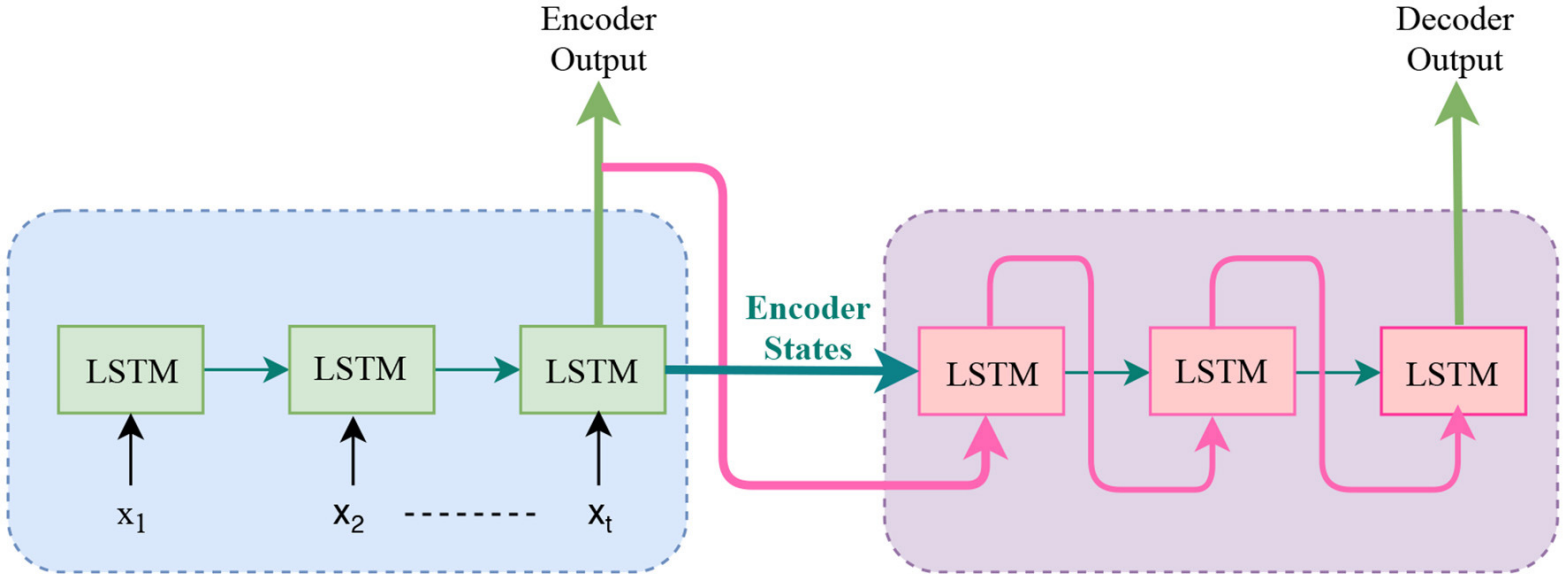
Sequence to Sequence model: This mode uses “encoder-decoder” and maps input sequence with “encoder states” and use it as initial state of the decoder.

A Seq2Seq model was first introduced by Sutskever et al. (2014) as a learning method for natural language processing. This translation application is like time series prediction because it has a sequential model and it also has sequential input-output pairs. In their method, they use a multi-layered LSTM to map the input sequence to a vector with a fixed dimension. Then this vector is used by another deep LSTM to decode the target sequence from that vector. The sequence to sequence model consists of two blocks of LSTMs, which are incorporated into an encoder and decoder block, as shown in Figure 5. Their main result was that on an English to French translation tasks from the WMT-14 dataset, they produced a good result compared with other methods.

Mariet and Kuznetsov (2019) provided a theoretical study for time series prediction with sequence to sequence models. They found the *generalization bound* of sequence to sequence models for time series prediction. Furthermore, they proposed a measurement based on the sequence data properties to determine whether the Seq2Seq model can be successful. Seq2Seq models are also used extensively for the dynamic, spatial-temporal characteristic of multivariate time series data (Zhu and Laptev, 2017; Du et al., 2018; Yang et al., 2019; Salinas et al., 2020). Another application of the sequence to sequence model is in video and image captioning (Venugopalan et al., 2015; Yang et al., 2017).

#### 3.3.5. GAN Model

Generative Adversarial Network (GAN) is a kind of neural network which was introduced by Goodfellow et al. (2014) as a framework to estimate a generative model using an adversarial operation. This structure consists of two neural networks. First, a generative network produces new data based on the distribution of a training set. Second, a discriminative model evaluates the probability that a sample was drawn from it. Therefore, in time series prediction, it can generate new time series samples with the same distribution. GANs are quite new and were introduced and found suitable for unsupervised learning (Radford et al., 2015), deep reinforcement learning (Ho and Ermon, 2016) and semi-supervised learning (Donahue et al., 2016). There is an extensive exploration in time series prediction using GAN. Zhang et al. (2019) compare GAN with baseline models from ARIMA. They also compare shallow and deep LSTMs with GAN for stock market price predictions. Based on the result of this paper, the best performance is with a shallow LSTM; however, GAN has an acceptable performance. ForGAN is a model based on a conditional GAN with an LSTM/GRU layer and was proposed by Koochali et al. (2019) as a novel approach for forecasting future values. Another GAN model for time series prediction introduced by Zec et al. (2019). This model is a recurrent conditional GAN and uses LSTMs both as the generator and discriminator to express the long-term dependencies in time series. In this approach, they trained the network using the Seq2Seq method. Furthermore, Yoon et al. (2019) proposed the TimeGAN framework to capture the temporal dynamics of the time series data. They compared their novel network with Conditional and Recurrent GAN. Recently GAN-based architectures have become more popular for sequential data due to the generative property of these models. However, most papers and research in the time series prediction field still employ RNNs or LSTM.

## 4. Discussion

In this paper, we reviewed the time delay problem in the teleoperation. In the first section, we reviewed the early approaches and studies in this field. Most of the early methods focused on understanding and estimating the delay. Some work concentrated on the design of systems to reduce the computation load. Next, we focused on reviewing solutions for the time delay problem, which has the potential be used to help mitigate issues with time delay. We surveyed two major areas for time series prediction, which included both statistical and neural network approaches. In Table 1, we compare all the main methods for time series prediction covered here.

**Table 1:** Comparison of the selected methods reviewed in this paper.

**Method**	**Linearity**	**Stationarity**	**Advantage**	**Disadvantage**
AR	Linear	Stationary	Applies lags and shifts of historical data; simplicity	Unsuitable for nonlinear and non-stationary signals; susceptibility to noise
MA	Linear	Stationary	Reliable result for stationary signal	Unsuitable for nonlinear and non-stationary signals; can only predict one step in the future
ARMA	Linear	Stationary	AR model with a MA to improve the result	Not appropriate for long-term prediction and non-stationary signals
ARIMA	Nonlinear	Non-stationary	Promotes ARMA by adding an integral term to handle non-stationery	Unsuitable for long-term prediction, cannot fully capture the non-linearity
RNN	Nonlinear	Non-stationary	Utilizes the saved information in the past via feedback	Long-term dependencies; vanishing gradient and exploding gradient
LSTM	Nonlinear	Non-stationary	Solves long-term dependencies and vanishing gradient in RNN	More prone to overfitting, longer training time and require more memory to train because of more parameters
Seq2Seq	Nonlinear	Non-stationary	Better mapping of input and output relationships; suitable for nonlinear time series	More parameters in comparison with LSTM, slower learning
GAN	Nonlinear	Non-stationary	Generative inheritance to learn the distribution of time series, shows good results for temporal setting	Because of adversarial component it cannot guarantee to capture the dependencies

The general advantage of the statistical methods is that they do not require training data and tend to be relatively simple methods with clear implementation avenues. Though the statistical methods are the traditional solutions for time series prediction, they cannot model all non-stationary signals. There are some extensions of the ARMA model that can handle non-stationary signals like ARIMA. If the data has seasonality, then other models such as SARIMA/SARIMAX were introduced. However, all these techniques deal with a specific kind of non-stationarity. Another disadvantage of these statistical methods is that they are not suitable for modeling complex tasks. The longer the dependencies, the more difficult to predict. These techniques are more appropriate for short-term predictions. In contrast with statistical models, neural network methods can be used to describe the data without necessarily knowing the distribution of the data. Moreover, by introducing LSTM networks, the literature shows that we can model complicated time series data taking into account many of the past dependencies. It is also possible that neural networks could be used in an adaptive way to change behaviors as more data is available. In this paper, we reviewed new neural network architectures for time series prediction. These methods consist of Seq2Seq and GAN models. The main advantage of these methods over traditional NN methods is that they are able to take inputs of variable sizes. These methods are promising for time series prediction due to the capability of their architecture to capture the time series distributions.

In the various neural network-based approaches, the relation between input and output is not clearly understood. It is modeled mostly as a black box of weights. In order to have a greater understanding of the mechanism of this transfer function, we point to a new approach by using the concepts of *information theory*. *Information theory* allows us to explain better and perhaps control the complex relationships. This idea is beginning to be used in deep learning by Shwartz-Ziv and Tishby (2017) and followed by Alemi et al. (2016) for variational autoencoders.

Readers are directed to the following repository, where several of the promising techniques described here are implemented https://github.com/parinazfa/Recent-Trends-in-Teleoperation-Time-DelayMitigation.git.

## 5. Conclusion

The time delay problem in teleoperation systems is an important challenge; therefore, several approaches from control-based to deep learning methods have been reviewed in this paper. This survey paper was divided into two parts: first, we covered traditional approaches to mitigating time delay in teleoperation. These methods included stability analysis and predictive methods for teleoperation systems. These methods focused on understanding and estimating the delay. Second, we covered time series prediction methods for mitigating time delay. These methods included the model of user intent or the system using time series prediction techniques. We reviewed statistical and NN based methods which are applied to mitigating time delay in teleoperation systems. We also reviewed some other deep learning models which may prove to beneficial for mitigation of time delay in teleoperation systems.

We believe that the new machine learning methods for time series prediction open a promising avenue for solving the time delay problem in teleoperation systems. A system that can predict the short-term future may be able to compensate for the time delay. It could also be able to adapt to new data and change its mode based on the situation.

Time series prediction opens a new avenue for safety. A related application to teleoperation is based on the prediction capability of time series approaches. If one can predict the immediate future when teleoperating (even in real-time teleoperation), dangerous future events can be mitigated to create safer systems. For example, in telesurgery systems, time series prediction along with real-time surface mapping and registration can warn the surgeon (or even stop or dampen movements) before dangerous tool's movements lead to bleeding, etc. Similarly, in other teleoperation systems, the map of the remote site along with an intelligent overwatch system, can inform the operator about possible dangers and inadvertent movements. Hence, solving the time delay issue with predictive technologies will also have these other important and related applications.

## Ethics Statement

Written informed consent was obtained from the individual(s) for the publication of any potentially identifiable images or data included in this article.

## Author Contributions

HY contributed to the AI sections related to LSTM, GAN, and general editing of the document. All authors contributed to the article and approved the submitted version.

## Conflict of Interest

The authors declare that the research was conducted in the absence of any commercial or financial relationships that could be construed as a potential conflict of interest.

## Abbreviations

AIC: akaike information criterion
AR: auto-regressive
ARIMA: auto-regressive integrated moving average
ARMA: auto-regressive moving average
convLSTM: convolutional LSTM
GAN: generative adversarial networks
GRU: gated recurrent unit
HSMM: hidden semi-Markov model
HWES: holt winter's exponential smoothing
LQG: linear quadratic gaussian
LSTM: long short-term memory networks
LSTM-VAE: LSTM and variational auto-encoder
MA: moving average
MIMO: multiple-input and multiple-output
NN: neural network
RNN: recurrent neural networks
SARIMA: seasonal autoregressive integrated moving-average
SARIMAX: seasonal autoregressive integrated moving-average with exogenous
SES: simple exponential smoothing
Seq2Seq: sequence-to-sequence
SMLR: sparse multivariate linear regressive
VARMA: vector autoregression moving-average
VARMAX: vector autoregression moving-average with exogenous regressors.
